# Differential effects of larval and adult nutrition on female survival, fecundity, and size of the yellow fever mosquito, *Aedes aegypti*

**DOI:** 10.1186/s12983-021-00395-z

**Published:** 2021-03-09

**Authors:** Jiayue Yan, Roumaissa Kibech, Chris M. Stone

**Affiliations:** Illinois Natural History Survey, University of Illinois at Urbana-Champaign, 1816 S. Oak St, Champaign, IL 61820 USA

**Keywords:** Nutritional stress, Mosquito longevity, Survival curves, Egg number, Wing length, Hazard ratios

## Abstract

**Background:**

The yellow fever mosquito, *Aedes aegypti*, is the principal vector of medically-important infectious viruses that cause severe illness such as dengue fever, yellow fever and Zika. The transmission potential of mosquitoes for these arboviruses is largely shaped by their life history traits, such as size, survival and fecundity. These life history traits, to some degree, depend on environmental conditions, such as larval and adult nutrition (e.g., nectar availability). Both these types of nutrition are known to affect the energetic reserves and life history traits of adults, but whether and how nutrition obtained during larval and adult stages have an interactive influence on mosquito life history traits remains largely unknown.

**Results:**

Here, we experimentally manipulated mosquito diets to create two nutritional levels at larval and adult stages, that is, a high or low amount of larval food (HL or LL) during larval stage, and a good and poor adult food (GA or PA, represents normal or weak concentration of sucrose) during adult stage. We then compared the size, survival and fecundity of female mosquitoes reared from these nutritional regimes. We found that larval and adult nutrition affected size and survival, respectively, without interactions, while both larval and adult nutrition influenced fecundity. There was a positive relationship between fecundity and size. In addition, this positive relationship was not affected by nutrition.

**Conclusions:**

These findings highlight how larval and adult nutrition differentially influence female mosquito life history traits, suggesting that studies evaluating nutritional effects on vectorial capacity traits should account for environmental variation across life stages.

**Supplementary Information:**

The online version contains supplementary material available at 10.1186/s12983-021-00395-z.

## Background

The yellow fever mosquito (Diptera: Culicidae), *Aedes aegypti* (Linnaeus, 1762), is the principal vector of several arthropod-borne viruses (i.e., arboviruses) such as dengue, yellow fever, chikungunya and Zika, which continue to impose a heavy burden on public health globally [1–5]. Dengue virus (DENV), for example, is estimated to cause 390 million cases of human infection each year, 96 million of which have clinical manifestations [6]. These arboviruses have been re-emerging in many regions and expanding their ranges across the globe, partly due to urbanization and subsequent expansion of the distribution of *Ae. aegypti* [7]. Given their medical importance, the vectorial capacity of mosquitoes has been an important focus of study [8, 9]. Vectorial capacity is simply an equation that isolates the entomological parameters from the basic reproduction number of a vector-borne disease (e.g., malaria [10]), often focusing on those parameters that can be measured under field conditions. It is a tremendously useful measure of transmission potential, which can guide implementation of control measures and increase our understanding of risk. Yet understanding the causes of variation in transmission potential between areas requires in-depth knowledge of the vector traits that influence vectorial capacity in a single locality. Mosquito life history traits, such as body size, survival and fecundity, can directly or indirectly influence mosquito population dynamics and vectorial capacity. For example, Alto et al [11] found that smaller-sized *Ae. aegypti* females were more susceptible to DENV infection and more likely to disseminate it than their larger counterparts. Longevity is a key competent of vectorial capacity as vectors must survive long enough to allow pathogens to replicate to a high level before the virus can be disseminated in subsequent bites [12]. Longevity and fecundity additionally affect the lifetime reproductive output of mosquitoes, and thereby influence local mosquito abundance, which also features as a parameter in the vectorial capacity equation. Despite the importance of life history traits, however, relatively few studies have examined how these traits can be influenced by the different environments experienced by mosquitoes across their developmental stages.

As an organism with a complex life cycle, mosquitoes experience highly distinct habitats from larval to adult stages and environmental factors may play a critical role in their fitness and performance [13]. The environment experienced by larvae may affect adult phenotypes through so called “carry-over effects” [14, 15]. For example, larval competition, food quantity and temperature have been reported to affect adult survival, size, longevity and vector competence [16–20]. At the same time, the environment experienced by adults, such as food quality/availability, or air temperature and the level of humidity, can also directly affect their life history traits and vector competence [21, 22]. Nonetheless, how environmental factors in both larval and adult stages may interactively affect life history traits or various aspects of mosquito behavior remains largely unknown (but see [23] for the influence of both larval and adult nutrition on mosquito biting persistence).

Nutrition is one of the environmental factors that affects all mosquito life history traits as it fuels development, growth, and performance. During the larval stage, microorganisms and particulate organic detritus are major nutritional resources and their abundance is readily affected by environmental changes, such as rainfall, competition, and predators of larvae [24]. As a container breeder, larval populations of *Ae. aegypti* can be regulated by nutritional stress derived from food limitation in the aquatic habitat [25]. After emergence, adult *Ae. aegypti* start foraging for food from terrestrial habitats nearby. Most mosquito species rely on plant sugars as an energy supply, while female mosquitoes require vertebrate blood as a nutritional resource for egg production. Previous studies suggested that female *Ae. aegypti* rarely feed on sugar [26] and that feeding on human blood alone may provide them with a fitness advantage [27, 28]. However, sugar-feeding by female *Ae. aegypti* may not be as unusual as thought previously, as support for frequent sugar-feeding in certain environments has been reported [29–31], and this propensity has been used to design attractive toxic sugar baits for *Ae. aegypti* control [32, 33]. Like larvae, adults may also be influenced by nutritional stress derived from changes in food quality (e.g., sugar concentration [34]). Both larval and adult nutritional stress has been shown to associate with adult survival, reproduction, and growth [35]. However, little is known about whether and how larval nutritional stress influences the effects of adult nutrition on life history traits.

Here we experimentally examined the potential interactive effects of larval (quantity) and adult (sucrose concentration) nutrition on survival and fecundity of adult female *Ae. aegypti*. To do that, we set up cohorts with two amounts of food during larval stages and two concentrations of sucrose solution during the adult stage and compared life history traits between different levels of nutritional treatments.

## Methods

### Mosquito rearing and treatments

**Fig. 1:**
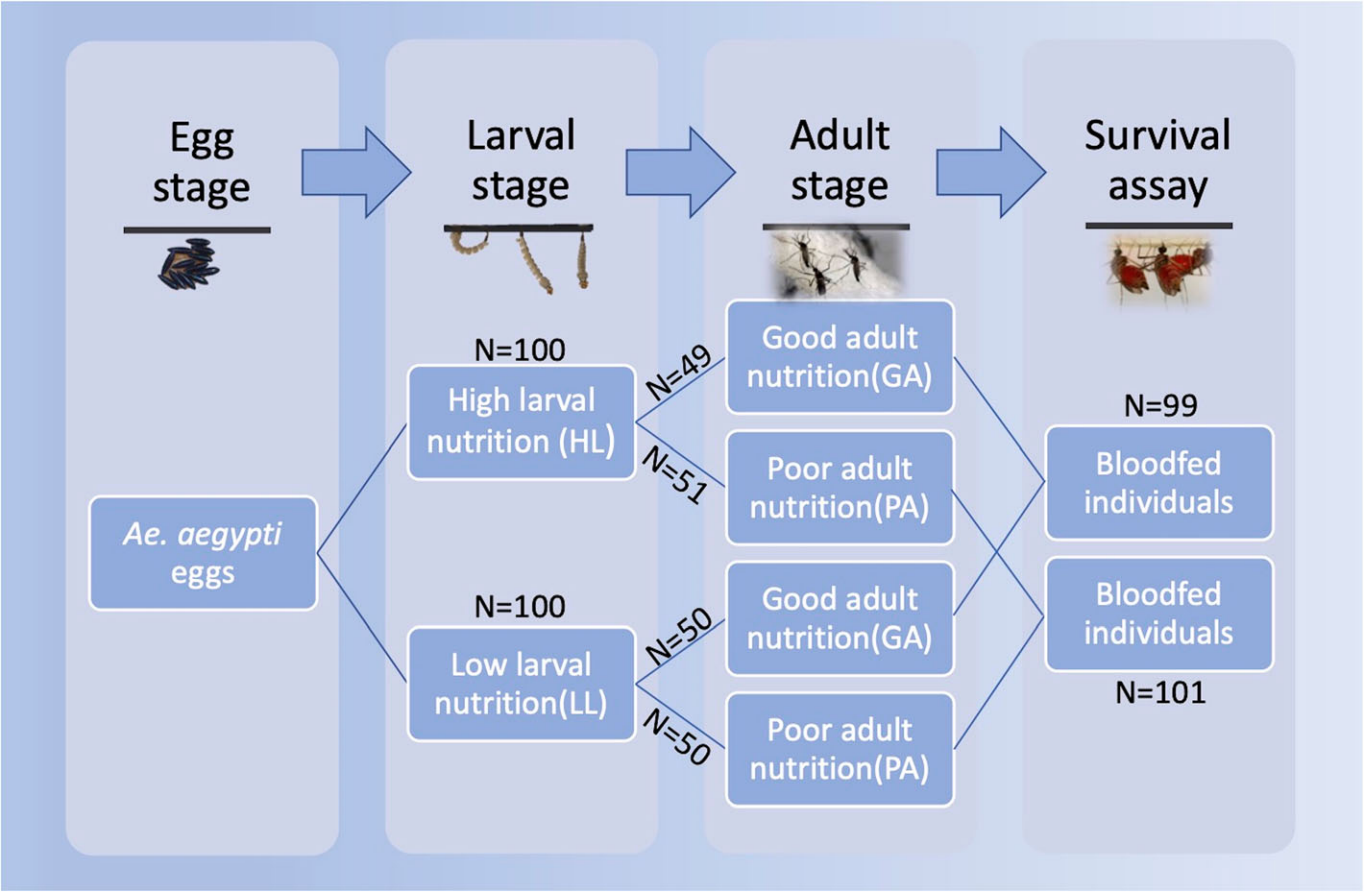
The schematic diagram of experimental design. High or low larval nutrition represents an access to larval food on days 2, 4, 5, and 6 post hatching or on days 2 and 6 post hatching; Good or poor adult nutrition represents an ad libitum access to 10% or 1% sucrose solution daily; Females were allowed to mate and take a blood meal before the start of the survival assay

All mosquitoes were cultured using the F_19_ generation of an *Ae. aegpyti* colony established from eggs collected in Key West, FL. Eggs were hatched overnight in an enamel pan (35 × 25 × 6 cm) filled with 500 mL of deionized (DI) water and 2 g of brain heart infusion (Difco Laboratories, Detroit, USA). To minimize potential effects of variation in larval density on mosquito fitness and performance [36], first-instar larvae were randomly counted and 100 of them were placed in each enamel pan filled with 500 mL of DI water. The larvae were reared under two nutritional regimes, following Joy et al [35] and Telang et al [37]: a well-nourished treatment where 100 mg of rabbit chow: lactalbumin: yeast (1:1:1) diet (Sigma-Aldrich, St. Louis, USA) was provided on days 2, 4, 5 and 6 post-hatching, representing high larval nutrition (hereafter HL); or a malnourished treatment where 100 mg of the same diet was provided only on days 2 and 6 post hatching, representing low larval nutrition (hereafter LL). The pupation rate for larvae reared under HL and LL was 94.5 and 89.9%, respectively, and no extreme death event was observed in any larvae-rearing pan. Eclosed adults from each larval nutritional treatment were maintained in paperboard cages (20.5 cm height × 18.5 cm diameter) and randomly assigned to one of two adult nutritional regimes with different food quality: a well-nourished treatment with ad libitum access to a 10% sucrose solution, representing good adult nutrition (hereafter GA); or a malnourished treatment with ad libitum access to 1% sucrose solution, representing poor adult nutrition (hereafter PA). Hence, there are two different levels of larval and adult nutrition, respectively (Fig. 1; Additional file 1). After keeping males and females together for 3–5 days to allow for mating, mosquitoes were cold-anesthetized at 4 °C and sexed on chilled Petri dishes using a stereomicroscope (Stemi DV4, Carl Zeiss AG, Jena, Germany). Females were retained in smaller paperboard cages (12 cm height × 11 cm diameter) with ad libitum access to the same adult nutritional treatments as above. Larvae and adults were kept in incubators (I-36VL, Geneva Scientific LLC, Fontana, USA) at 27 (±1) °C and 75 (±5) % relative humidity (RH) under a 12:12 h Light (L): Dark (D) photoperiod throughout the experiments.

### Bioassays and life history traits

Six-to-eight day old females were provided with access to bovine blood (Hemostat Laboratories, Dixon, USA) for 45 min via a Hemotek Membrane Feeding System (PS6, Hemotek Ltd., Blackburn, UK). Prior to the blood-feeding assay, these mosquitoes had been starved for 24 h by depriving them of sucrose solutions. From 24 to 12 h prior to the blood-feeding assay a cotton roll soaked with DI water was provided to them. Engorged mosquitoes were separated from unfed ones on chilled petri dishes after cold-anesthesia at 4 °C for 10 min. Fifty engorged individuals were randomly selected from each nutritional level and placed individually in small paperboard cages (5.5 cm height × 9 cm diameter) for life history assays. In each cage, a strip of seed germination paper was placed along the inner wall and kept moist daily from day 2 to 7 post blood-feeding to allow for oviposition. All caged individuals were provided with ad libitum access to either a 1% (PA) or 10% (GA) sucrose solution until death (see Fig. 1). Mortality of mosquitoes was checked daily and longevity was recorded as the number of days from blood-feeding to death (hereafter post-blood-feeding longevity). Immediately after the death of a mosquito, all the eggs inside a cage (including germination paper and all inner surface of the cage) were counted using a stereomicroscope. The measure of fecundity we recorded was the total number of eggs counted in a cage. Dead individuals were removed and stored at − 80 °C until their wing length could be measured, as a standard proxy for body size. Wing length was measured as the distance from the axial incision to the apical margin excluding the fringe of the scales [38]. The measurement of wing length was conducted using an inverted microscope (IX51, Olympus, Japan) and Olympus cellSens Entry 2.3 software.

### Statistical analyses

Two-way analysis of variance (ANOVA) was conducted to detect whether there were significant differences in post blood-feeding longevity, body size and fecundity between any two levels of larval nutrition, adult nutrition and their interaction. The normality of these three variables was examined in normal quantile plots. Outliers that exceeded the range of upper or lower whiskers in Tukey’s boxplots were removed before the two-way ANOVA [39, 40]. To further assess the effects of larval and adult nutritional stress on daily survival of mosquitoes, a survival analysis was performed using the R packages *survival* [41] and *survminer* [42] with the Kaplan-Meier Method and Log-Rank Test. A Cox Proportional Hazards model (CPH) was fitted to assess the death risk of mosquitoes reared from different levels of the treatments. We also examined potential trade-offs between life history traits by performing linear regression analyses between each pair of traits. To further test whether nutritional treatment influences the significant relationship between any two of the three life history traits from the above linear regressions, analysis of covariance (ANCOVA) was performed to compare the regression slopes of different levels of the treatments using the package *car* [43]. Statistical analyses were carried out in R software v. 3.6.3 [44].

## Results

**Fig. 2:**
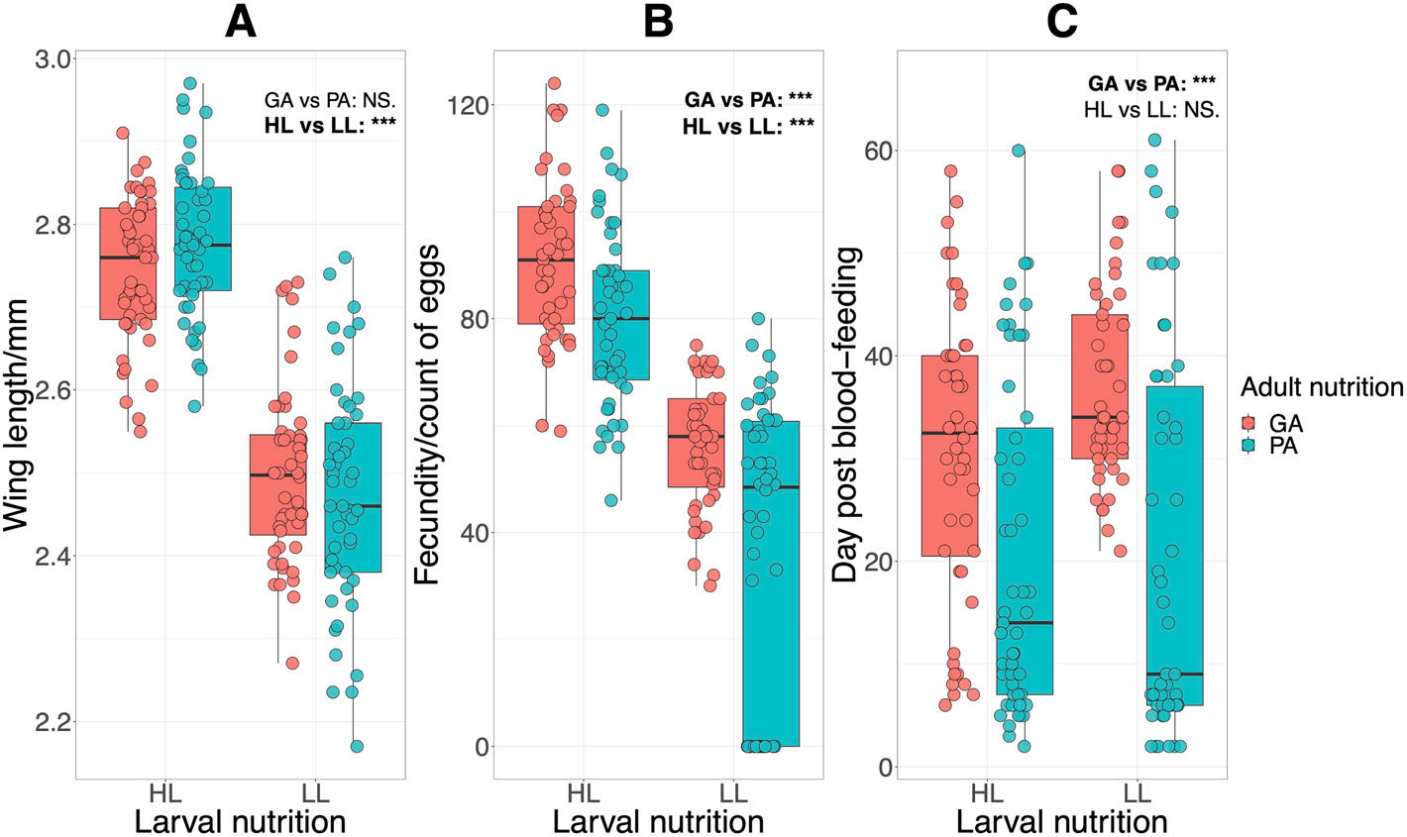
Differences in wing length (**a**), fecundity (**b**) and survival (**c**) of *Aedes aegypti* between treatment levels. HL: high larval nutrition, LL: low larval nutrition, GA: good adult nutrition and PA: poor adult nutrition, vs: versus, NS.: not significant, ***: *p* < 0.001. The line within each box indicates the median and the edges of each box the first (Q1) and third (Q3) quartiles; the whiskers extend over 1.5 times the interquartile range. A significant difference in wing length between two levels of larval nutrition was found (two-way ANOVA, for HL vs LL: F_1, 193_ = 336.77, *p* < 0.001, for GA vs PA: F_1, 193_ = 0.09, *p* = 0.77). For fecundity, both the comparisons between two levels of larval and adult nutrition were significant (two-way ANOVA, for HL vs LL: F_1, 182_ = 194.25, *p* < 0.001; for GA vs PA: F_1, 182_ = 29.43, *p* < 0.001). There was a significant difference in survival between two levels of adult nutrition (two-way ANOVA, for GA vs PA: F_1, 190_ = 36.44, *p* < 0.001, for HL vs LL: F_1, 190_ = 1.67, *p* = 0.20)

The mean wing length of mosquitoes from high larval nutrition (HL), low larval nutrition (LL), good adult nutrition (GA) and poor adult nutrition (PA) was 2.76 ± 0.01 SE mm, 2.49 ± 0.01 SE mm, 2.62 ± 0.02 SE mm and 2.63 ± 0.02 SE mm, respectively (Table 1). There was significant difference between HL and LL (two-way ANOVA, F_1, 193_ = 336.77, *p* < 0.001; Fig. 2a), indicating that larval food quantity significantly affected adult size. As wing length is fixed in adults, no difference was found between adult nutritional levels (two-way ANOVA, F_1, 193_ = 0.09, *p* = 0.76; Fig. 2a). The interaction between larval and adult nutrition was not significant (two-way ANOVA, F_1, 193_ = 3.46, *p* = 0.06).

**Table 1:** Mean wing length, fecundity and survival of *Aedes aegypti* by different levels of treatment

Treatment	Wing length	Fecundity	Survival
Larval nutrition			
HL	2.76 ± 0.01 SE	85.34 ± 1.75 SE	25.47 ± 1.61 SE
LL	2.49 ± 0.01 SE	45.37 ± 2.54 SE	28.28 ± 1.74 SE
Adult nutrition			
GA	2.62 ± 0.02 SE	73.70 ± 2.33 SE	33.67 ± 1.31 SE
PA	2.63 ± 0.02 SE	57.29 ± 3.29 SE	20.57 ± 1.72 SE

Abbreviations used in the table listed as following. *HL* high larval nutrition, *LL* low larval nutriton, *GA* good adult nutrition, *PA* poor adult nutrition, *SE* standard error. Mean wing length is recorded to 2 decimal places in mm and measured as described in the main text. Mean fecundity is represented by the number of eggs laid. Mean survival is the number of days that the individual lived post-blood-feeding

The mean fecundity of mosquitoes from HL, LL, GA and PA was 85.34 ± 1.75 SE, 45.37 ± 2.54 SE, 73.70 ± 2.33 SE and 57.29 ± 3.29 SE, respectively (Table 1). There was a significant difference between HL and LL (two-way ANOVA, F_1, 182_ = 194.25, *p* < 0.001; Fig. 2b), and between GA and PA (two-way ANOVA, F_1, 182_ = 29.43, *p* < 0.001; Fig. 2b), indicating that both the larval and adult diets affected mosquito egg-laying. However, there was no statistically significant interaction between larval and adult nutrition on fecundity (two-way ANOVA, F_1, 182_ = 2.94, *p* = 0.08), suggesting that the effects of larval and adult nutrition on fecundity were additive rather than synergistic.

**Fig. 3:**
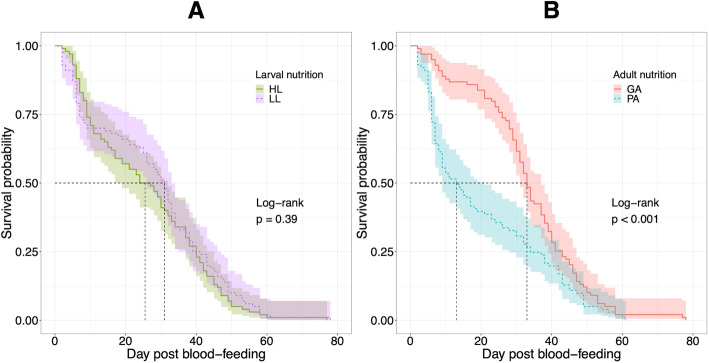
Survival curves between nutritional levels of larval nutrition (**a**) and adult nutrition (**b**). Survival probabilities were estimated by Kaplan-Meier method and shadow areas represent 95% confidence intervals. HL: high larval nutrition, LL: low larval nutrition, GA: good adult nutrition, PA: poor adult nutrition. The dotted line represents day at median survival for each nutritional level (HL = 25.5, LL = 31.0, GA = 33.0, and PA = 13.0). There was a significant difference in survival probability between GA and PA (Log-rank p < 0.001), while the difference between HL and LL was not significant (Log-rank *p* = 0.39)

**Fig. 4:**
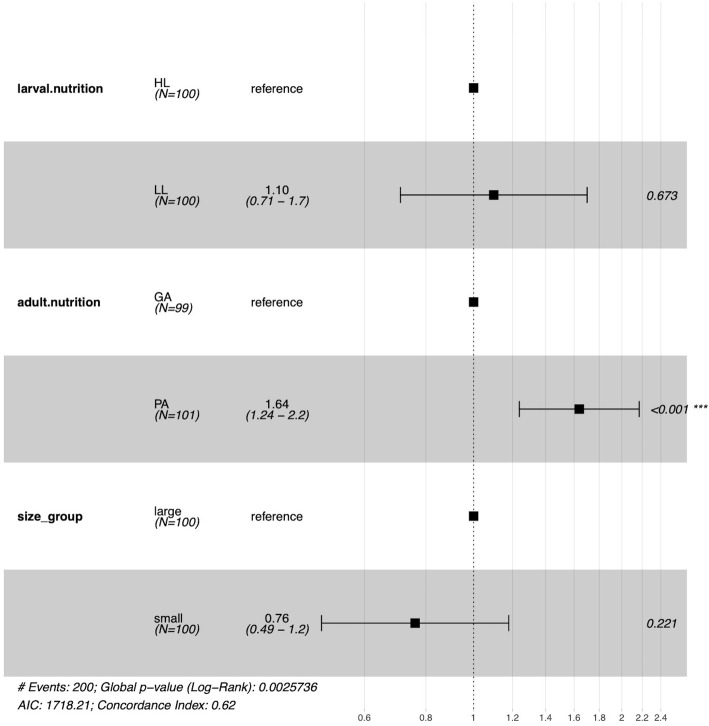
Hazard ratios for mosquitoes from different nutritional levels and body size (wing length). Cox Proportional-Hazards model showed that poor adult nutrition increased the death risk of mosquitoes (hazard ratio 1.64, *p* < 0.001, GA as reference), while the effects of larval nutrition (*p* = 0.67) and body size (*p* = 0.22) were not significant

The mean post blood-feeding longevity of mosquitoes from HL, LL, GA and PA was 25.47 ± 1.61 SE d, 28.28 ± 1.74 SE d, 33.67 ± 1.31 SE d and 20.57 ± 1.72 SE d, respectively (Table 1). There was a significant difference between GA and PA (two-way ANOVA, F_1, 190_ = 36.44, *p* < 0.001; Fig. 2c), indicating that adult food quality significantly affected adult longevity. No significant effect was found between HL and LL (two-way ANOVA, F_1, 190_ = 1.67, *p* = 0.20; Fig. 2c) nor in larval and adult nutritional interaction (two-way ANOVA, F_1, 190_ = 2.00, *p* = 0.16), indicating that larval food quantity did not affect adult survival. Survival curves also showed a significant difference between GA and PA (Log-rank *p* < 0.001; Fig. 3) and no difference between HL and LL (Log-rank *p* = 0.39; Fig. 3). The CPH model including larval nutrition, adult nutrition and wing length (body size) as covariates indicated that poor adult nutrition increased the death risk of mosquitoes (hazard ratio 1.64, *p* < 0.001, GA as reference; Fig. 4), while the effects of larval nutrition (*p* = 0.67; Fig. 4) and body size (*p* = 0.22; Fig. 4) were not significant..

**Fig. 5:**
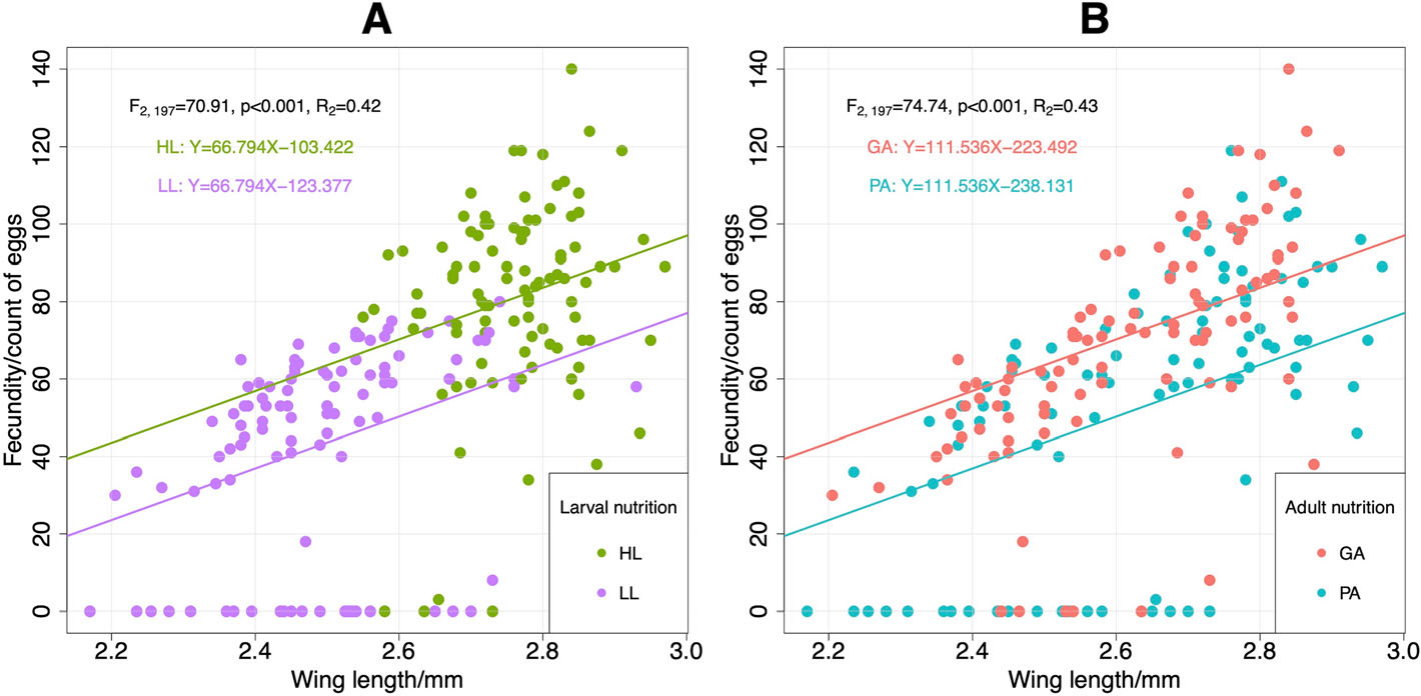
Regression relationship between fecundity and wing length of mosquitoes from larval (**a**) and adult nutrition (**b**). HL: high larval nutrition, LL: low larval nutrition, GA: good adult nutrition, PA: poor adult nutrition. Analysis of covariance (ANCOVA) showed that the positive relationship between fecundity and wing length did not change at different nutritional levels of larval (slope = 66.79, *p* < 0.001) or adult nutrition (slope = 111.54, *p* < 0.001). The effects of nutritional treatments on fecundity after controlling for the effect of body size (wing length) were significant (larval nutrition: F_2_,_197_ = 70.91, *p* < 0.001, R_2_ = 0.42; adult nutrition: F_2_,_197_ = 74.74, *p* < 0.001, R_2_ = 0.43)

There was a positive correlation between fecundity and wing length (linear regression using fecundity and wing length as dependent and explanatory variable respectively: estimate ± SE = 110.18 ± 10.04, t = 10.98, *p* < 0.001, *R*^*2*^ = 0.38). The slope of this positive relationship did not differ between two levels of larval (ANCOVA, slope = 66.79, *p* < 0.001; Fig. 5a) or adult nutrition (ANCOVA, slope = 111.54, *p* < 0.001; Fig. 5b). In addition, the effects of nutritional treatments on fecundity after controlling for the effect of body size (wing length) were significant (larval nutrition: F_2,197_ = 70.91, *p* < 0.001, *R*_*2*_ = 0.42; adult nutrition: F_2,197_ = 74.74, *p* < 0.001, *R*_*2*_ = 0.43). There was also a significantly positive relationship between mosquito fecundity and survival (estimate ± SE = 0.29 ± 0.13, t = 2.23, *p* = 0.027, *R*^*2*^ = 0.02), but no significant relationship was found between wing length and survival (estimate ± SE = − 0.001 ± 0.001, t = − 1.38, *p* = 0.17).

## Discussion

In this study we investigated how different quantities of larval and adult nutrition affect several key life history traits of adult female *Ae. aegypti* mosquitoes. We found that while adult size was influenced by larval nutrition, and survival was influenced only by adult nutrition, fecundity depended on both larval and adult nutrition.

The quantity of larval nutrition affected mosquito wing length, a proxy for body size, which is consistent with previous studies [37, 45, 46]. Body size has been suggested to be an important life history trait of mosquitoes because of its close connection to or correlation with other traits that influence fitness and susceptibility to infection and dissemination [10]. In our study, however, the effect of body size was only noticeable on fecundity.

Mosquitoes feeding on a 10% sucrose solution had a significantly greater longevity than their counterparts feeding on a 1% sucrose solution, regardless of larval nutrition. Similarly, Briegel et al [47] found that higher sucrose concentrations (0.5–50%) extend the survival time of *Ae. aegypti*, probably because higher concentrations allow for greater increases in energy reserves. Larval nutrition, in our case, had no significant influence on adult survivorship. A negative effect of increased larval nutrition on adult *Ae. aegypti* longevity has been reported by previous studies [35]. However, larval competition for nutrition (i.e., reduced larval nutrition) can also reduce adult *Ae. aegypti* longevity under certain conditions (e.g., under stress related to low humidity) [18]. Opposite effects of larval nutrition on adult longevity have thus been reported within this species. Similar contradictory results have also been reported in other mosquito species (e.g., *Anopheles gambiae* [45, 48]), indicating that populations of different genetic origins will likely have different life history responses to nutritional stress, though this is an area for further research. Besides differences in the genetic background of different mosquito populations used for these experiments, it is possible that the differences in outcomes between studies could be caused by the methodological diversities among studies, such as larval food quantity and quality used, as well as larval density or habitat characteristics. Some studies used fish food or liver powder-based diet as larval nutrition [49, 50], while others used microorganisms as the natural diet for larvae [51], which further handicaps the direct comparison of results between studies. It is also possible that effects of larval nutrition on longevity are only expressed when mosquitoes are placed in stressful conditions, though the current study suggests that low sucrose availability at least does not induce that outcome. Besides these differences in methodology, whether a female is mated or not could also mediate the effect of larval nutrition on insect lifespan. May et al [52], for example, demonstrated that virgin female *Drosophila melanogaster* reared on poor larval nutrition lived longer than their counterparts reared on more nutritious resources, while there was no influence of the amount of larval food level on the longevity of mated females. We did not vary the level of exposure to males in this study, but this would be an interesting avenue for further research.

Both larval and adult nutrition significantly affected mosquito fecundity. Both mosquitoes that were exposed to the high food regime as larvae and had access to a 10% sucrose solution as adults laid more eggs than those that had access to the lower levels of nutrition. This is in accordance with Vantaux et al [46] who found that adult *An. coluzzii* reared under low levels of larval food were significantly less fecund. With regard to adult nutrition, it is known that blood meal quantity and source can influence mosquito fecundity [35, 53–55], while intake of carbohydrates can also influence egg production [56, 57]. Energy reserves can be a more decisive factor for fecundity than protein, for example, Mostowy and Foster [58] found that egg number of *Ae. aegypti* does not correspond to blood meal size but instead closely associated with the level of energetic reserves at the time of blood-feeding. Plant-sugar meals are shunted to the ventral diverticulum, or crop, which, when full can compete for space in the midgut for blood meals and thereby reduce blood meal intake and fecundity [58]. In our study, where mosquitoes were starved for 1 day before blood feeding, crops would have likely been considerably emptied [56], and the effect of adult nutrition levels on fecundity did not appear to depend on the nutritional reserves obtained during the larval stages, suggesting rather an additive effect of reserves on fecundity. Traditionally fecundity of a mosquito was measured as the total number of oviposited eggs and retained follicles [59]. However, oviposited eggs as a proxy for fecundity is also commonly used in recent studies [60–62], especially for those carried out with survival experiment, where retained follicles could likely be resorbed by mosquitoes later [63].

We did not detect any trade-offs between mosquito survival, size and fecundity. Trades-offs between life history traits of organisms have often been observed as a result of a limited resource that has to be allocated to growth, development and performance [64]. Here, we found an expected positive relationship between wing length and fecundity (i.e., larger-sized mosquitoes can lay larger egg clutches), and we found that egg numbers also depended on both larval and adult nutrition even after controlling for body size. However, we also found a positive relationship between fecundity and survival, indicating that longer-lived mosquitoes could also lay more eggs. Future work could explore whether other traits (e.g., related to immune function or metabolic detoxification [37]) do provide evidence of a trade-off in relation to mosquito nutrition.

While the individual effects of larval and adult nutrition on mosquito life history traits are well established, the underlying mechanisms for such effects are seldomly examined (but see [65]) and thus, poorly understood. In other insects such as *D. melanogaster*, the insulin/insulin-like growth factor signaling pathway has been regarded as a sensor of the insect’s nutritional status and a regulator of lifespan and reproduction [66–69]. Future effort could focus on this pathway in order to reveal the mechanism underlying the effect of nutrition on mosquito longevity and fecundity.

## Conclusions

In conclusion, mosquito larval and adult nutrition may have differential effects on their life history traits. While larval food quantity and adult food quality influence body size and survival respectively, both quantity and quality jointly affect mosquito fecundity. This has potentially important ramifications for our understanding of population dynamics and vectorial capacity of mosquitoes, in that both larval and adult environments should be considered when tracking factors influencing mosquito fitness and performance.

## Supplementary Information

**Additional file 1 **Wing length, fecundity and survival of *Aedes aegypti* reared at different nutritional levels. Abbreviations used in the table listed as following. ID: mosquito identity, HL: high larval nutrition, LL: low larval nutrition, GA: good adult nutrition, PA: poor adult nutrition. Wing length is recorded to 3 decimal places in mm and measured as described in the main text. Fecundity is represented by the number of eggs laid. Survival is the number of days that the individual lived post-blood-feeding.

## Data Availability

All data generated or analyzed during this study are included in this published article and its supplementary information files.
