# Supplementary material for: Differential effects of larval and adult nutrition on female survival, fecundity, and size of the yellow fever mosquito, Aedes aegypti
Source: Front Zool. 2021 Mar 9;18:10. doi: 10.1186/s12983-021-00395-z (PMC7941737; doi:10.1186/s12983-021-00395-z)
Supplement: Supplementary file 1 — Additional file 1 Wing length, fecundity and survival of Aedes aegypti reared at different nutritional levels. Abbreviations used in the table listed as following. ID: mosquito identity, HL: high larval nutrition, LL: low larval nutrition, GA: good adult nutrition, PA: poor adult nutrition. Wing length is recorded to 3 decimal places in mm and measured as described in the main text. Fecundity is represented by the number of eggs laid. Survival is the number of days that the individual lived post-blood-feeding. [file 12983_2021_395_MOESM1_ESM.doc]

**Differential effects of larval and adult nutrition on female survival, fecundity, and size of the yellow fever mosquito *Aedes aegypti* – *Frontiers in Zoology* – Yan *et al*. 2020– INHS, UIUC – Email:** [**jiayue@illinois.edu**](mailto:jiayue@illinois.edu)

**Additional file 1**

| ID | Larval nutrition | Adult nutrition | Wing length | Fecundity | | Survival |
| --- | --- | --- | --- | --- | --- | --- |
| 1 | LL | PA | 2.74 | 80 | | 8 |
| 2 | LL | PA | 2.65 | 0 | | 6 |
| 3 | LL | PA | 2.255 | 0 | | 49 |
| 4 | LL | PA | 2.41 | 49 | | 49 |
| 5 | LL | PA | 2.6 | 66 | | 7 |
| 6 | LL | PA | 2.46 | 69 | | 54 |
| 7 | LL | PA | 2.53 | 0 | | 6 |
| 8 | LL | PA | 2.7 | 0 | | 2 |
| 9 | LL | PA | 2.585 | 73 | | 19 |
| 10 | LL | PA | 2.49 | 43 | | 49 |
| 11 | LL | PA | 2.56 | 61 | | 7 |
| 12 | LL | PA | 2.385 | 53 | | 43 |
| 13 | LL | PA | 2.395 | 0 | | 2 |
| 14 | LL | PA | 2.435 | 0 | | 5 |
| 15 | LL | PA | 2.51 | 68 | | 5 |
| 16 | LL | PA | 2.5 | 61 | | 58 |
| 17 | LL | PA | 2.5 | 53 | | 6 |
| 18 | LL | PA | 2.68 | 65 | | 21 |
| 19 | LL | PA | 2.34 | 49 | | 6 |
| 20 | LL | PA | 2.345 | 33 | | 7 |
| 21 | LL | PA | 2.415 | 53 | | 32 |
| 22 | LL | PA | 2.535 | 0 | | 43 |
| 23 | LL | PA | 2.38 | 43 | | 16 |
| 24 | LL | PA | 2.37 | 0 | | 5 |
| 25 | LL | PA | 2.38 | 48 | | 6 |
| 26 | LL | PA | 2.36 | 0 | | 7 |
| 27 | LL | PA | 2.675 | 0 | | 2 |
| 28 | LL | PA | 2.455 | 62 | | 56 |
| 29 | LL | PA | 2.67 | 75 | | 33 |
| 30 | LL | PA | 2.45 | 0 | | 2 |
| 31 | LL | PA | 2.445 | 53 | | 6 |
| 32 | LL | PA | 2.56 | 0 | | 5 |
| 33 | LL | PA | 2.235 | 36 | | 32 |
| 34 | LL | PA | 2.49 | 0 | | 8 |
|  |  |  |  |  | |  |
|  | **Additional file 1(continued)** | | | | |  |
| ID | Larval nutrition | Adult nutrition | Wing length | Fecundity | | Survival |
| 35 | LL | PA | 2.455 | 65 | | 38 |
| 36 | LL | PA | 2.59 | 59 | | 6 |
| 37 | LL | PA | 2.51 | 51 | | 18 |
| 38 | LL | PA | 2.42 | 58 | | 14 |
| 39 | LL | PA | 2.57 | 50 | | 7 |
| 40 | LL | PA | 2.46 | 64 | | 34 |
| 41 | LL | PA | 2.17 | 0 | | 38 |
| 42 | LL | PA | 2.28 | 0 | | 26 |
| 43 | LL | PA | 2.235 | 0 | | 2 |
| 44 | LL | PA | 2.31 | 0 | | 38 |
| 45 | LL | PA | 2.525 | 0 | | 2 |
| 46 | LL | PA | 2.76 | 60 | | 39 |
| 47 | LL | PA | 2.315 | 31 | | 9 |
| 48 | LL | PA | 2.58 | 61 | | 61 |
| 49 | LL | PA | 2.52 | 40 | | 9 |
| 50 | LL | PA | 2.93 | 58 | | 26 |
| 51 | HL | PA | 2.865 | 70 | | 5 |
| 52 | HL | PA | 2.94 | 96 | | 6 |
| 53 | HL | PA | 2.58 | 0 | | 2 |
| 54 | HL | PA | 2.83 | 111 | | 9 |
| 55 | HL | PA | 2.88 | 89 | | 6 |
| 56 | HL | PA | 2.63 | 77 | | 43 |
| 57 | HL | PA | 2.75 | 86 | | 7 |
| 58 | HL | PA | 2.67 | 60 | | 7 |
| 59 | HL | PA | 2.83 | 86 | | 49 |
| 60 | HL | PA | 2.655 | 3 | | 32 |
| 61 | HL | PA | 2.7 | 59 | | 15 |
| 62 | HL | PA | 2.775 | 107 | | 34 |
| 63 | HL | PA | 2.66 | 56 | | 42 |
| 64 | HL | PA | 2.77 | 60 | | 49 |
| 65 | HL | PA | 2.84 | 102 | | 30 |
| 66 | HL | PA | 2.82 | 68 | | 7 |
| 67 | HL | PA | 2.625 | 82 | | 24 |
| 68 | HL | PA | 2.68 | 58 | | 13 |
| 69 | HL | PA | 2.78 | 81 | | 9 |
| 70 | HL | PA | 2.85 | 63 | | 8 |
| 71 | HL | PA | 2.85 | 103 | | 30 |
| 72 | HL | PA | 2.785 | 71 | | 14 |
| 73 | HL | PA | 2.935 | 46 | | 17 |
| 74 | HL | PA | 2.72 | 71 | | 10 |
|  | **Additional file 1(continued)** | | | |  | |
| ID | Larval nutrition | Adult nutrition | Wing length | Fecundity | | Survival |
| 75 | HL | PA | 2.725 | 79 | | 3 |
| 76 | HL | PA | 2.77 | 98 | | 37 |
| 77 | HL | PA | 2.675 | 87 | | 13 |
| 78 | HL | PA | 2.73 | 0 | | 45 |
| 79 | HL | PA | 2.72 | 75 | | 5 |
| 80 | HL | PA | 2.7 | 98 | | 47 |
| 81 | HL | PA | 2.86 | 85 | | 9 |
| 82 | HL | PA | 2.95 | 70 | | 23 |
| 83 | HL | PA | 2.85 | 108 | | 5 |
| 84 | HL | PA | 2.75 | 89 | | 42 |
| 85 | HL | PA | 2.8 | 73 | | 6 |
| 86 | HL | PA | 2.855 | 70 | | 5 |
| 87 | HL | PA | 2.78 | 34 | | 28 |
| 88 | HL | PA | 2.72 | 72 | | 60 |
| 89 | HL | PA | 2.85 | 56 | | 23 |
| 90 | HL | PA | 2.775 | 67 | | 9 |
| 91 | HL | PA | 2.76 | 119 | | 6 |
| 92 | HL | PA | 2.9 | 89 | | 17 |
| 93 | HL | PA | 2.73 | 93 | | 10 |
| 94 | HL | PA | 2.775 | 88 | | 11 |
| 95 | HL | PA | 2.725 | 100 | | 42 |
| 96 | HL | PA | 2.81 | 69 | | 43 |
| 97 | HL | PA | 2.79 | 84 | | 11 |
| 98 | HL | PA | 2.715 | 64 | | 15 |
| 99 | HL | PA | 2.785 | 63 | | 17 |
| 100 | HL | PA | 2.78 | 80 | | 4 |
| 101 | HL | PA | 2.97 | 89 | | 45 |
| 102 | LL | GA | 2.365 | 42 | | 45 |
| 103 | LL | GA | 2.54 | 71 | | 51 |
| 104 | LL | GA | 2.5 | 51 | | 35 |
| 105 | LL | GA | 2.545 | 49 | | 78 |
| 106 | LL | GA | 2.56 | 70 | | 39 |
| 107 | LL | GA | 2.44 | 0 | | 58 |
| 108 | LL | GA | 2.37 | 51 | | 29 |
| 109 | LL | GA | 2.365 | 34 | | 31 |
| 110 | LL | GA | 2.27 | 32 | | 32 |
| 111 | LL | GA | 2.55 | 56 | | 32 |
| 112 | LL | GA | 2.385 | 45 | | 46 |
| 113 | LL | GA | 2.38 | 65 | | 29 |
| 114 | LL | GA | 2.58 | 71 | | 32 |
|  | **Additional file 1(continued)** | | |  | |  |
| ID | Larval nutrition | Adult nutrition | Wing length | Fecundity | | Survival |
| 115 | LL | GA | 2.71 | 70 | | 43 |
| 116 | LL | GA | 2.455 | 63 | | 53 |
| 117 | LL | GA | 2.39 | 58 | | 34 |
| 118 | LL | GA | 2.64 | 72 | | 37 |
| 119 | LL | GA | 2.41 | 47 | | 31 |
| 120 | LL | GA | 2.41 | 55 | | 41 |
| 121 | LL | GA | 2.47 | 18 | | 43 |
| 122 | LL | GA | 2.73 | 8 | | 6 |
| 123 | LL | GA | 2.5 | 53 | | 33 |
| 124 | LL | GA | 2.205 | 30 | | 39 |
| 125 | LL | GA | 2.72 | 70 | | 28 |
| 126 | LL | GA | 2.545 | 71 | | 33 |
| 127 | LL | GA | 2.39 | 53 | | 49 |
| 128 | LL | GA | 2.54 | 65 | | 26 |
| 129 | LL | GA | 2.45 | 60 | | 30 |
| 130 | LL | GA | 2.58 | 59 | | 3 |
| 131 | LL | GA | 2.45 | 44 | | 25 |
| 132 | LL | GA | 2.445 | 57 | | 53 |
| 133 | LL | GA | 2.54 | 0 | | 3 |
| 134 | LL | GA | 2.67 | 60 | | 26 |
| 135 | LL | GA | 2.725 | 72 | | 44 |
| 136 | LL | GA | 2.58 | 63 | | 58 |
| 137 | LL | GA | 2.59 | 75 | | 34 |
| 138 | LL | GA | 2.435 | 53 | | 47 |
| 139 | LL | GA | 2.54 | 72 | | 39 |
| 140 | LL | GA | 2.35 | 40 | | 23 |
| 141 | LL | GA | 2.43 | 40 | | 28 |
| 142 | LL | GA | 2.45 | 50 | | 31 |
| 143 | LL | GA | 2.53 | 0 | | 2 |
| 144 | LL | GA | 2.52 | 62 | | 30 |
| 145 | LL | GA | 2.465 | 0 | | 21 |
| 146 | LL | GA | 2.495 | 62 | | 48 |
| 147 | LL | GA | 2.405 | 59 | | 33 |
| 148 | LL | GA | 2.5 | 46 | | 34 |
| 149 | LL | GA | 2.76 | 58 | | 34 |
| 150 | LL | GA | 2.45 | 41 | | 25 |
| 151 | LL | GA | 2.51 | 58 | | 46 |
| 152 | HL | GA | 2.845 | 76 | | 38 |
| 153 | HL | GA | 2.82 | 110 | | 40 |
| 154 | HL | GA | 2.78 | 76 | | 30 |
|  | **Additional file 1(continued)** | | |  | |  |
| ID | Larval nutrition | Adult nutrition | Wing length | Fecundity | | Survival |
| 155 | HL | GA | 2.84 | 140 | | 21 |
| 156 | HL | GA | 2.91 | 119 | | 29 |
| 157 | HL | GA | 2.845 | 94 | | 38 |
| 158 | HL | GA | 2.635 | 0 | | 32 |
| 159 | HL | GA | 2.715 | 80 | | 24 |
| 160 | HL | GA | 2.71 | 97 | | 33 |
| 161 | HL | GA | 2.675 | 86 | | 7 |
| 162 | HL | GA | 2.825 | 91 | | 31 |
| 163 | HL | GA | 2.69 | 102 | | 9 |
| 164 | HL | GA | 2.685 | 41 | | 55 |
| 165 | HL | GA | 2.85 | 108 | | 47 |
| 166 | HL | GA | 2.825 | 92 | | 19 |
| 167 | HL | GA | 2.7 | 108 | | 46 |
| 168 | HL | GA | 2.775 | 83 | | 34 |
| 169 | HL | GA | 2.66 | 94 | | 10 |
| 170 | HL | GA | 2.68 | 72 | | 11 |
| 171 | HL | GA | 2.72 | 102 | | 50 |
| 172 | HL | GA | 2.775 | 98 | | 40 |
| 173 | HL | GA | 2.71 | 82 | | 27 |
| 174 | HL | GA | 2.62 | 73 | | 50 |
| 175 | HL | GA | 2.72 | 100 | | 40 |
| 176 | HL | GA | 2.68 | 89 | | 37 |
| 177 | HL | GA | 2.77 | 96 | | 21 |
| 178 | HL | GA | 2.82 | 87 | | 33 |
| 179 | HL | GA | 2.68 | 74 | | 6 |
| 180 | HL | GA | 2.585 | 92 | | 45 |
| 181 | HL | GA | 2.565 | 78 | | 40 |
| 182 | HL | GA | 2.77 | 119 | | 77 |
| 183 | HL | GA | 2.795 | 85 | | 28 |
| 184 | HL | GA | 2.875 | 38 | | 38 |
| 185 | HL | GA | 2.55 | 76 | | 30 |
| 186 | HL | GA | 2.84 | 60 | | 8 |
| 187 | HL | GA | 2.76 | 99 | | 24 |
| 188 | HL | GA | 2.81 | 86 | | 41 |
| 189 | HL | GA | 2.79 | 101 | | 47 |
| 190 | HL | GA | 2.72 | 79 | | 37 |
| 191 | HL | GA | 2.625 | 77 | | 41 |
| 192 | HL | GA | 2.76 | 75 | | 29 |
| 193 | HL | GA | 2.8 | 118 | | 37 |
| 194 | HL | GA | 2.84 | 80 | | 8 |
|  | **Additional file 1(continued)** | | |  | |  |
| ID | Larval nutrition | Adult nutrition | Wing length | Fecundity | | Survival |
| 195 | HL | GA | 2.73 | 59 | | 58 |
| 196 | HL | GA | 2.78 | 101 | | 53 |
| 197 | HL | GA | 2.605 | 93 | | 9 |
| 198 | HL | GA | 2.865 | 124 | | 19 |
| 199 | HL | GA | 2.705 | 89 | | 16 |
| 200 | HL | GA | 2.81 | 104 | | 7 |
